# Age-related mortality risk in cycling trauma: analysis of the National Trauma Databank 2017–2023

**DOI:** 10.1186/s40621-024-00558-6

**Published:** 2025-01-24

**Authors:** Marta A. W. Rowh, Taylor A. Giller, John N. Bliton, Randi N. Smith, Tim P. Moran

**Affiliations:** 1 Department of Emergency Medicine, Emory University, 531 Asbury Circle, Annex Building Suite N340, Atlanta, GA 30322 USA; 2 Jamaica Hospital Medical Center, 8900 Van Wyck Expy, Richmond Hill, NY 11418 USA; 3 Department of Surgery, Emory University, 69 Jesse Hill Jr. Dr. SE, Atlanta, GA 30303 USA

**Keywords:** Cycling, Geriatrics, Trauma, Mortality

## Abstract

**Background:**

Cycling promotes health but carries significant injury risks, especially for older adults. In the U.S., cycling fatalities have increased since 1990, with adults over 50 now at the highest risk. As the population ages, the burden of cycling-related trauma is expected to grow, yet age-specific factors associated with mortality risk remain unclear. This study identifies age-specific mortality risk thresholds to inform targeted public health strategies.

**Methods:**

We conducted a cross-sectional analysis of the National Trauma Data Bank (NTDB) data (2017–2023) on non-motorized cycling injuries. A total of 185,960 records were analyzed using logistic regression with splines to evaluate the relationship between age and mortality risk. The dataset was split into training (80%) and testing (20%) sets. Age thresholds where mortality risk changed were identified, and models were adjusted for injury severity, comorbidities, and helmet use.

**Results:**

The median patient age was 43 years (IQR 20–58). Four key age thresholds (12, 17, 31, and 69) were identified, with the largest mortality increase after age 69. Our model achieved an AUC of 0.93, surpassing traditional age cutoff models, with 84.6% sensitivity and 88.0% specificity.

**Conclusions:**

Age is a significant predictor of mortality in cycling trauma, with marked increases in risk during adolescence and for adults over 69. These findings underscore the need for age-targeted interventions, such as improved cycling infrastructure for teens and enhanced safety measures for older adults. Public health initiatives should prioritize these vulnerable age groups to reduce cycling-related mortality.

## Introduction

Cycling activity, i.e. non-motorized bicycles, improves functional status, cognition, and general well-being [1–3], but also is associated with risk of traumatic injury. Fatality rates for cycling have been increasing in the United States (U.S.) since 1990, a trend which has not been seen in Europe [4]. In both regions, the risk of mortality per distance traveled is greater when cycling than when traveling by car [5–7].

As the median age of the U.S. increases [8] the share of cyclists who are older adults has increased as well. Over the prior decade, the average age of cycling fatalities has increased from 41 to 47 and cyclists aged 50–54 now have the highest fatality rate [9]. In the coming decades, this trend is expected to continue. By 2050, approximately 20% of Americans are expected to be over 65 years of age [10] and the percentage of patients in trauma registries considered elderly is expected to have increased from 30 to 40% [11].

Age has long been considered a risk for mortality in traumatic injury, but debate remains over what age defines elderly and what criteria are most predictive of increased risk [12]. Traditionally, the trauma literature has defined age over 65 as a categorical risk factor for increased mortality, but this may not apply to all older adult populations or for all injury types [13]. For example, Stitzel et al. evaluated the age which optimized the prediction of mortality following a motor vehicle crash and found cut points ranging between 47 and 58 [14]. Similar work has not been conducted in a traumatically injured cycling population.

Studies demonstrate improvements in disease prevention, chronic disease management, functional status, and overall well-being among older adults who remain physically active [2]. In the context of cycling, several studies have identified reductions in cardiovascular risk, the incidence of various cancers, and all-cause mortality [15–18]. Previous research has shown that increased frailty and decreased functional status are associated with a higher risk of traumatic injury; however, less attention has been given to active older adult populations [19, 20]. Because of these differences, it is possible that age cut offs identified in populations injured during sedentary forms of transportation will not apply individuals injured while cycling.

The present study sought to empirically determine the age threshold(s) at which the risk of mortality increases in patients who suffer a traumatic injury during cycling using a cross-validation approach. Understanding the age at which cycling mortality risk increases can inform the targeting of public health interventions.

## Methods

### Study design

This project is a national cross-sectional study of patient encounters documented in the U.S. National Trauma Data Bank (NTDB) during the period January 1, 2017 to December 31, 2023. The NTDB, the largest U.S. trauma database, includes over six million patient records collected from over 900 trauma centers across the U.S [21]. For inclusion in the NTDB, patients must sustain a traumatic injury that resulted in Emergency Medical Services (EMS) transfer, medical evaluation, admission, or death at any level I or level II trauma center (or a level III/IV center that provides data voluntarily) [22]. Individual hospitals define their trauma service activation protocols, but generally any patient who sustains a possibly life-threatening injury, or need for surgery, will result in trauma activation [23]. All entries resulting from a bicycling injury, as identified by International Classification of Diseases, Tenth Revision, Clinical Modification (ICD-10-CM) codes were included in the study. Motorized bicycles of any kind were excluded. This resulted in 185,960 entries.

### Statistical analysis

Categorical variables were described using frequencies and percentages. Continuous/scale variables were described using medians and interquartile ranges (IQR). Logistic regressions were used to evaluate the relationship between age and mortality risk. Because this analysis is inherently exploratory (i.e. there are a large number of possible non-linear relationships), the use of significance testing would result in a high rate of false positives. Instead, we took a cross-validation based approach. This involved splitting the data into two sets: the training set (*N* = 148,768, 80%) and the testing/validation set (*N* = 37,192, 20%). The training set was used to explore the relationship between age and mortality and build, or “train”, a model.

The relationship between age and mortality was evaluated by transforming age into linear spline representations. Rather than fitting a single linear relationship across the entire range of age, this method fits several piecewise curves across a limited range of age by fitting “knots” at specific ages. Thus, the relationship between age and mortality can change at different levels of age. For example, if a knot were placed at 65, this would indicate that the odds ratio relating age to mortality differed between those greater than 65 and those under 65.

Within the training set, the number and location of knots were set using leave-one-out cross validation. For each combination of knots, we evaluated the binomial deviance:

$$\text{Binomial Deviance} = \sum -2\ast [{Y}_{i} \ast ln(pi)+(1-Yi) \ast ln(1-pi)]$$

Where Yi is the observed outcome for patient i (Y = 0 if the patient survived; Y = 1 if the patient died), Pi is the model predicted probability of mortality for patient i, and ln is the natural logarithm. Binomial deviance is a measure of how poor a prediction is; thus, the set of splines with the lowest binomial deviance provides the best fit for the data.

All models were adjusted and included the following variables: age, gender, race, injury severity score, ICD code (V10 through V19), alcohol screening, whether a helmet was worn, and comorbidities (attention-deficit/hyperactivity disorder (ADHD), alcoholism, anticoagulant, bleeding, chemotherapy, cirrhosis, congenital diseases, chronic obstructive pulmonary disease (COPD), cerebrovascular accident (CVA), dementia, diabetes, disseminated cancer, functional independence, congestive heart failure (CHF), hypertension, myocardial infarction (MI), peripheral artery disease (PAD), psychiatric, renal, smoking, steroid, other substance abuse, other documented diseases). Frequencies for medical comorbidities are presented in Supplementary Table 1 in the appendix.

Once a set of splines was selected, it could then be evaluated using confirmatory methods in the testing set. Importantly, the testing set could not influence the model building process and using the testing set to evaluate the model allows us to determine how the model generalizes to a new sample. Three models were fit in the testing set: a baseline model which included all variables except for age, a model which included all variables and an age threshold of 65, and a model which included all variables and the best-fitting splines for age. These models were then compared using χ^2^ goodness of fit tests. To further evaluate the models, we present Receiver Operating Characteristic (ROC) curves and the area under the ROC curves (AUC). Across all relevant data points, < 1% of values were missing (Supplemental Table 2 in the appendix). Missing data were imputed using fully conditional specification [24]. Analyses were conducted using R (v 4.3; R Core Team).

## Results

### Sample characteristics

**Fig. 1:**
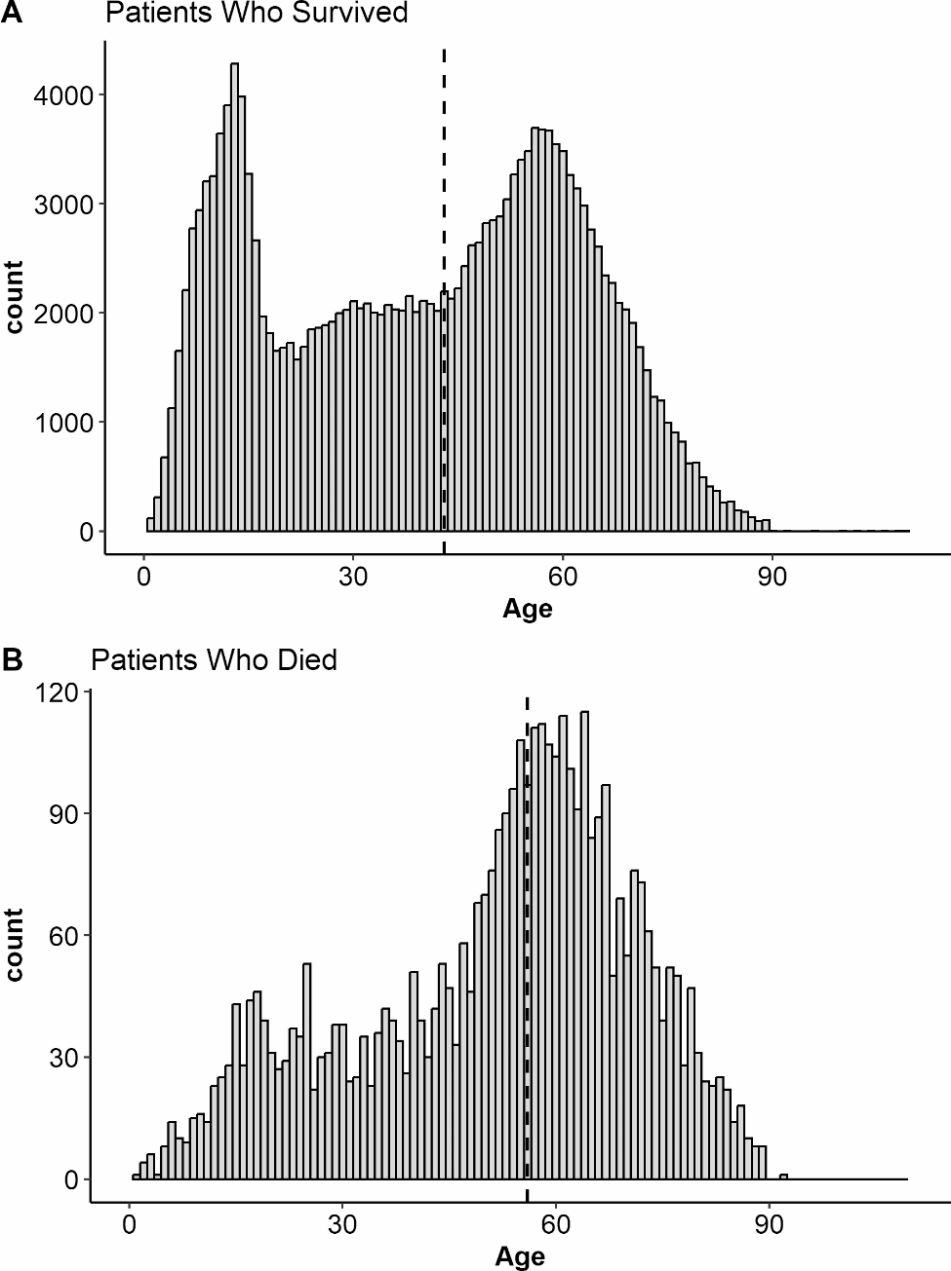
Histogram for age

The sample consisted of 185,960 patients with a median age of 43 (IQR 20–58). A histogram of sample ages is presented in Fig. 1. Females made up about 19% of the total sample and White patients made up about 75% of the sample. The median Injury Severity Score (ISS) for the sample was 9 (IQR 4–13), in the moderate injury range. Approximately 11% of the sample were severely injured (ISS: 16–24) and 6% were critically injured (ISS > 24). 4,080 (2.2%) experienced a fatal injury from cycling (Table 1). Helmet use was observed in 34% of the overall sample, compared to 19% among patients who died. The (NTDB) categorizes alcohol screening results as positive, negative, or not tested. Within this sample, the majority of patients (55%) were not tested, 10% of survivors and 13% of the deceased had positive alcohol screening results. The most common ICD codes associated with these injuries were V18 (45.6%; Pedal cycle rider injured in non-collision transport accident), V13 (31.5%; Pedal cycle rider injured in collision with car, pick-up truck or van), V19 (9.4%; Pedal cycle rider injured in other and unspecified transport accidents), and V17 (8.5%; Pedal cycle driver injured in collision with fixed or stationary object in nontraffic accident).

**Table 1:** Patient Characteristics

**Characteristic**	**Survived** (N = 181,880)	**Died** (N = 4,080)	**Total** (N = 185,960)
**Age**, M (IQR)	43 (20 – 58)	56 (39 – 66)	43 (20 – 58)
**Sex**, N (%)			
Female	35,519 (19.53)	512 (12.55)	36,031 (19.38)
Male	146,272 (80.42)	3,566 (87.4)	149,838 (80.58)
Non-Binary/Trans	89 (0.05)	2 (0.05)	91 (0.05)
**Race**, N (%)			
Black	18,440 (10.14)	504 (12.35)	18,944 (10.19)
White	137,894 (75.82)	2,853 (69.93)	140,747 (75.69)
Asian	5,214 (2.87)	130 (3.19)	5,344 (2.87)
Other	5,771 (3.17)	138 (3.38)	5,909 (3.18)
**Hispanic**, N (%)	24,272 (13.35)	613 (15.02)	24,885 (13.38)
**Injury Severity Score**, M (IQR)	9 (4 – 12)	30 (21 – 38)	9 (4 – 13)
**Helmet Use**, N (%)	62,431 (34.33)	779 (19.09)	63,210 (33.99)
**Mechanism of Injury / ICD**, N (%)			
V10: Collision with pedestrian or animal	1,376 (0.76)	18 (0.44)	1,394 (0.75)
V11: Collision with other pedal cycle	4,858 (2.67)	42 (1.03)	4,900 (2.63)
V12: Collision with two- or three- wheeled motor vehicle	772 (0.42)	42 (1.03)	814 (0.44)
V13: Collision with car, pick-up truck or van in traffic accident	55,800 (30.68)	2,716 (66.57)	58,516 (31.47)
V14: Collision with heavy transport vehicle or bus	1,518 (0.83)	192 (4.71)	1,710 (0.92)
V15: Collision with railway train or railway vehicle	88 (0.05)	14 (0.34)	102 (0.05)
V16: Collision with other nonmotor vehicle in nontraffic accident	428 (0.24)	2 (0.05)	430 (0.23)
V17: Collision with fixed or stationary object in nontraffic accident	15,614 (8.58)	146 (3.58)	15,760 (8.47)
V18: Noncollision transport accident	84,295 (46.35)	555 (13.6)	84,850 (45.63)
V19: Injured in unspecified traffic accident	17,131 (9.42)	353 (8.65)	17,484 (9.4)
**Alcohol Screening**, N (%)			
Negative	63,388(34.9)	1,783 (43.7)	65,171 (35)
Not Tested	100,335 (55.2)	1,774 (43.5)	102,109 (54.9)
Positive	18,157 (9.98)	523 (12.82)	18,680 (10.05)
**Data Split**, N (%)			
Training Set	145,494 (79.99)	3,274 (80.25)	148,768 (80)
Testing Set	36,386 (20.01)	806 (19.75)	37,192 (20)

**Fig. 2:**
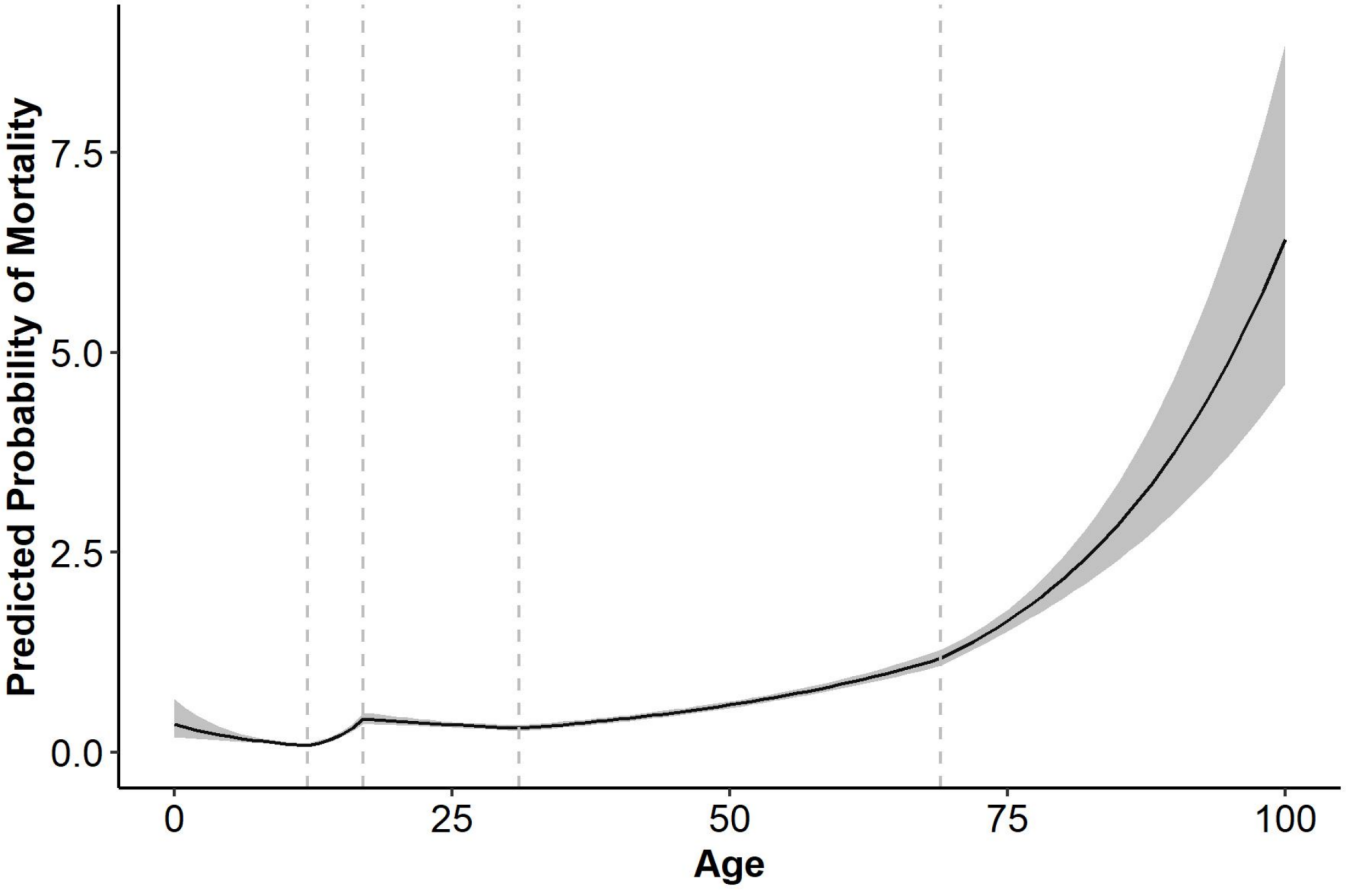
The best fitting relationship between age and mortality

With respect to the primary research question, the model building process identified four knot locations which resulted in the best fit. They were placed at the ages 12, 17, 31, and 69. When evaluated in the training subset, the model with these knots fit the data significantly better than both the baseline model (*p* < .001) and the model with a threshold at 65 (*p* < .001). The best fitting model is presented in Fig. 2. The knot locations indicate that the risk of mortality slightly decreases between the ages of 0 and 12, increases between 12 and 17, decreases between 17 and 31, slowly increases between 31 and 69, and then increases at a greater rate after age 69. Odds ratios within age ranges are presented in Table 2. Note that while age 12–17 was associated with the largest odds ratio, that age range is relatively small. Thus, the absolute increase in mortality risk is relatively small (Fig. 2). The largest absolute increase in mortality was observed after age 69 (Fig. 2).

**Table 2:** Odds ratio between age and mortality for each spline

Age Range	Odds Ratio	95% CI	*p*
<12	0.89	0.83–0.96	0.002
12 – 17	1.37	1.27–1.48	<0.001
17 – 31	0.98	0.96–0.99	0.003
31 – 69	1.04	1.03–1.05	<0.001
>69	1.06	1.04–1.08	<0.001

**Fig. 3:**
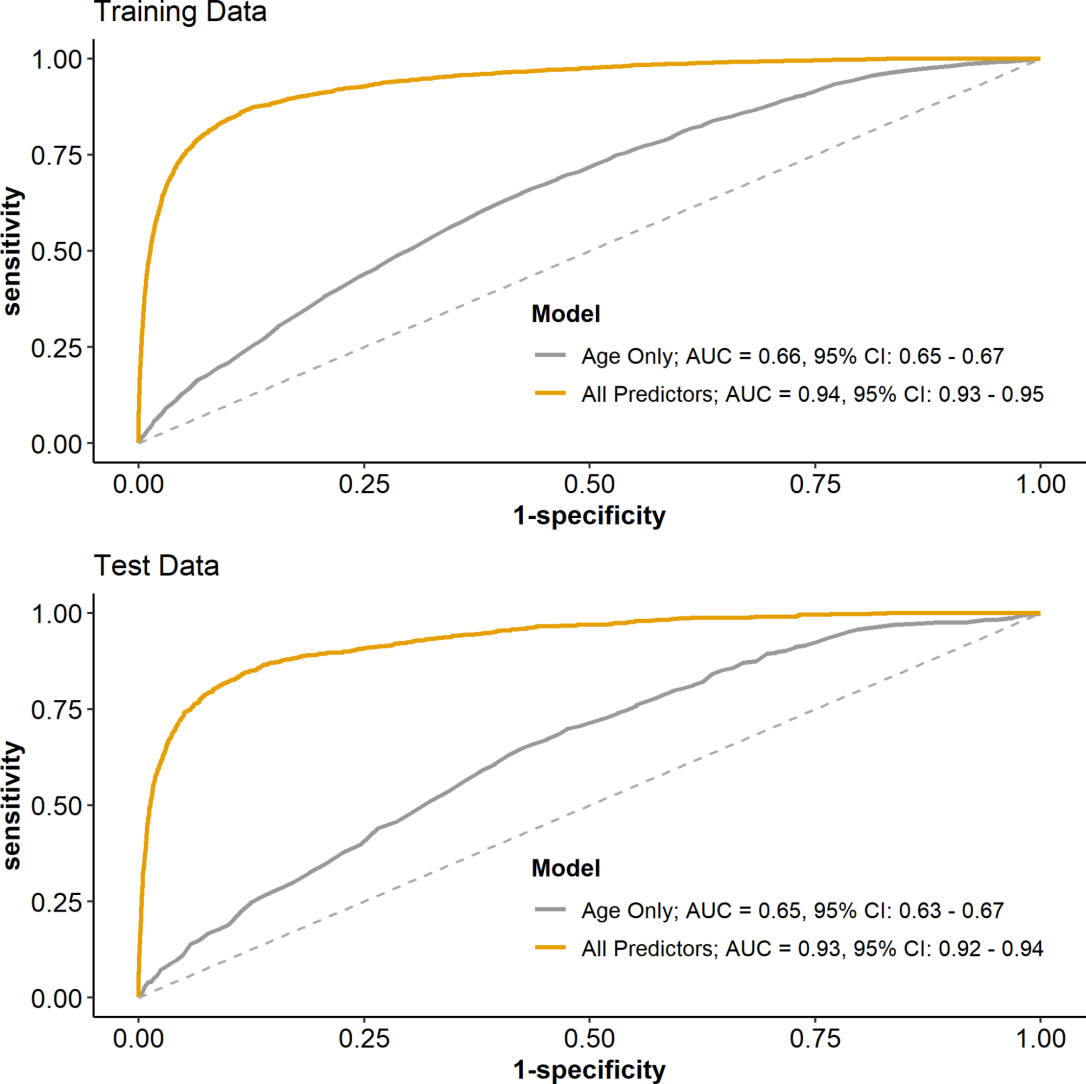
ROC curves for the prediction of mortality

The ROC for the training and test sets are presented in Fig. 3. The splines for age alone, developed in the training set, had an AUC of 0.65 (95% CI: 0.63–0.67) in the testing set. This increased to 0.93 (95% CI: 0.92–0.94) when the covariates are added to the model. The AUC for the model with an age threshold at 65, though high (AUC = 0.92, 95% CI: 0.91–0.93), was significantly lower than the AUC for the best fitting model (*p* = .0002). In terms of classification accuracy, the best fitting model had a sensitivity of 84.6%, and specificity of 88.0%, and an overall accuracy of 87.9%.

## Discussion

This study represents the first comprehensive analysis of age as a risk factor for cycling-related mortality across the lifespan within a U.S. population. Previous cycling research has predominantly been conducted outside the U.S., where cycling infrastructure, such as dedicated bike lanes, patterns of injury, and collision types differ [25]. However, as cycling rates rise in the U.S., country-specific studies are essential to guide effective public health interventions.

Our findings indicate that age is an independent predictor of mortality following cycling-related trauma, but this relationship is nonlinear, with distinct inflection points at ages 12, 17, 31, and 69. Importantly, these cut points were identified in the training data set and significantly improved fit in the testing set, indicating that these cut points generalize beyond the training data. Additionally, these findings are independent of other known risk factors such as ISS, comorbidities (at least those included in the NTDB), and mechanism of injury.

There is an increased risk of mortality from cycling in the teenage years, from ages 12–17, likely driven by a combination of biological and behavioral factors, including increased physical strength, stamina, and greater independence from adult supervision. In our study, 72.3% of fatal cycling accidents involved collisions with motor vehicles—including 2- and 3-wheeled vehicles, heavy transport vehicles, and buses—highlighting the critical importance of implementing measures to separate cycling traffic from motor vehicles to reduce mortality in this vulnerable population. Although international efforts to promote safe active transport to school (e.g., walking and cycling) have largely focused on younger, elementary-aged children and remain understudied in the U.S. [26], evidence from a United Kingdom (U.K.)-based intervention demonstrated the effectiveness of promoting helmet use, high-visibility clothing, and route planning to avoid motor vehicle traffic, with behavior change persisting into adolescence [27]. These findings suggest the need to trial similar evidence-based approaches tailored for adolescents in the U.S. context. Our research underscores the importance of addressing this gap through targeted studies and public health strategies that account for the unique vulnerabilities and behaviors of this age group.

The largest absolute increase in risk occurred following the age of 69, consistent with previous literature that older adults have higher mortality risk after trauma. However, most prior studies of cycling in this age group have been completed internationally with unclear implications for U.S. populations [28–30]. There are, however, international efforts that should be adapted for the U.S. The “Safer Cycling in Older Age” (SiFAr), a randomized controlled study out of Germany, showed efficacy in decreasing cycling errors among adults over age 65 [31]. This emphasizes the need to adapt evidence-based, international risk mitigation strategies, to reduce mortality and improve recovery outcomes.

The 2020 World Health Organization (WHO) guidelines on cyclist safety for policymakers did not address age as a specific risk factor [32]. However, our data suggest that specific age groups have different mortality risk profiles. Public health initiatives to reduce cycling-related mortality should be tailored to the distinct risks faced by different age groups and future research should prioritize the development and evaluation of age-specific injury prevention strategies.

## Limitations

While offering novel insights into traumatic injury risks for older populations, there is no exposure data available in this study. Therefore, this population may be heterogeneous in types of cycling, which could give insights into appropriate risk mitigation strategies. A prospective study evaluating traumatic injury risk in those whose type of cycling and amount of cycling is measured directly would further clarify this association. Also, as with all estimates, knots that we identified are subject to sampling variation.

## Data Availability

The datasets generated and/or analysed during the current study are available in the National Trauma Data Bank.

## Appendix

**Supplemental Table 1:** Frequencies of Medical Comorbidities in the NTDS Cycling Sample

**Comorbidities**	** Survived**	**Died**	**Total **
ADHD	2630 (1.45)	20 (0.49)	2650 (1.43)
Alcoholism	4706 (2.59)	112 (2.75)	4818 (2.59)
Anticoagulant	2068 (1.14)	62 (1.52)	2130 (1.15)
Bleeding	478 (0.26)	10 (0.25)	488 (0.26)
Chemo	118 (0.06)	4 (0.1)	122 (0.07)
Cirrhosis	502 (0.28)	48 (1.18)	550 (0.3)
Congenital	400 (0.22)	12 (0.29)	412 (0.22)
COPD	1852 (1.02)	30 (0.74)	1882 (1.01)
CVA	446 (0.25)	16 (0.39)	462 (0.25)
Dementia	246 (0.14)	10 (0.25)	256 (0.14)
Diabetes	3602 (1.98)	114 (2.79)	3716 (2)
Disseminated cancer	128 (0.07)	2 (0.05)	130 (0.07)
Functional Independence	428 (0.24)	8 (0.2)	436 (0.23)
CHF	446 (0.25)	16 (0.39)	462 (0.25)
Hypertension	12620 (6.94)	280 (6.86)	12900 (6.94)
MI	264 (0.15)	2 (0.05)	266 (0.14)
Other	11202 (6.16)	202 (4.95)	11404 (6.13)
PAD	90 (0.05)	2 (0.05)	92 (0.05)
Mental/Personality	5982 (3.29)	98 (2.4)	6080 (3.27)
Renal	180 (0.1)	12 (0.29)	192 (0.1)
Smoking	15180 (8.35)	166 (4.07)	15346 (8.25)
Steroid	260 (0.14)	4 (0.1)	264 (0.14)
Substance Abuse	6600 (3.63)	112 (2.75)	6712 (3.61)

**Supplemental Table 2:** Frequency and Percentage of Missing Data by Variable

**Variable**	**Missing** N (%)
Age	425 (0.2)
Race	985 (0.5)
Sex	309 (0.2)
Ethnicity	4,990 (2.7)
ICD Code	0 (0)
Alcohol Screening	2,169 (1.2)
ISS	290 (0.2)
Disposition	6 (<0.1)
Comorbid Conditions	0 (0)
Helmet Use	0 (0)

## Abbreviations

ADHD: Attention Deficit Hyperactivity Disorder
AUC: Area Under the Curve
CHF: Congestive Heart Failure
COPD: Chronic Obstructive Pulmonary Disease
CVA: Cerebral Vascular Accident
EMS: Emergency Medical Services
ICD-10-CM: International Classification of Diseases, Tenth Revision, Clinical Modification
IQR: Interquartile Range
ISS: Injury Severity Score
MI: Myocardial Infarction
NTDB: National Trauma Data Bank
PAD: Peripheral Arterial Disease
ROC: Receiver Operating Characteristic
U.K.: United Kingdom
U.S.: United States
WHO: World Health Organization
